# Propagation of superconducting coherence via chiral quantum-Hall edge channels

**DOI:** 10.1038/s41598-017-11209-w

**Published:** 2017-09-08

**Authors:** Geon-Hyoung Park, Minsoo Kim, Kenji Watanabe, Takashi Taniguchi, Hu-Jong Lee

**Affiliations:** 1 Department of Physics, Pohang University of Science and Technology, Pohang, 790-784 Republic of Korea; 2 Advanced Materials Laboratory, National Institute for Materials Science, 1-1 Namiki, Tsukuba, 305-0044 Japan

## Abstract

Recently, there has been significant interest in superconducting coherence via chiral quantum-Hall (QH) edge channels at an interface between a two-dimensional normal conductor and a superconductor (N–S) in a strong transverse magnetic field. In the field range where the superconductivity and the QH state coexist, the coherent confinement of electron- and hole-like quasiparticles by the interplay of Andreev reflection and the QH effect leads to the formation of Andreev edge states (AES) along the N–S interface. Here, we report the electrical conductance characteristics via the AES formed in graphene–superconductor hybrid systems in a three-terminal configuration. This measurement configuration, involving the QH edge states outside a graphene–S interface, allows the detection of the longitudinal and QH conductance separately, excluding the bulk contribution. Convincing evidence for the superconducting coherence and its propagation via the chiral QH edge channels is provided by the conductance enhancement on both the upstream and the downstream sides of the superconducting electrode as well as in bias spectroscopy results below the superconducting critical temperature. Propagation of superconducting coherence via QH edge states was more evident as more edge channels participate in the Andreev process for high filling factors with reduced valley-mixing scattering.

## Introduction

An electron incident from a normal conductor (N) to a superconductor (S) undergoes retroreflection as a hole at the N–S interface, which is the Andreev reflection (AR)^1^. In particular, for a two-dimensional (2D) conducting system such as a 2D electron gas (2DEG) layer in contact with a superconductor, an exotic AR effect emerges in the quantum-Hall (QH) regime in a strong transverse magnetic field, where carrier transport occurs through incompressible edge states while the bulk states are insulating^2, 3^. Here, the AR effect can be suppressed due to pair breaking for a spin-singlet superconductor. Even in a strong magnetic field, however, survival of the superconducting proximity effect by the AR at an N–S interface has been reported^4, 5^. From a quasiclassical point of view, such a nontrivial AR allows an incident electron and a reflected hole to form into an alternating skipping orbit along the N–S interface. Quantum mechanically this special bound state, called the Andreev edge state (AES)^6–9^, is considered to be the coherently superposed paired state of Andreev-reflected electrons and holes.

The coexistence of the QH edge states and the superconductivity can be realised as one reaches the QH regime in an N layer at sufficiently low magnetic field to allow the survival of the superconductivity in an S electrode. This requires the N layer to have high carrier mobility with a mean free path longer than the magnetic length (*l*_B_) and a good superconducting proximity contact as well at the 2DEG–S interface. 2DEG systems have been used in earlier studies to attain this high carrier mobility in a normal conducting layer; however, this often leaves a pronounced potential barrier at the interface^4, 10, 11^. This leads to suppression of the AR probability and a corresponding zero-bias peak in the interfacial differential resistance^12^. In contrast, graphene has been shown to provide a good electrical contact with diverse materials including conventional metallic superconductors^13–15^. Thus, graphene can be used to form various types of hybrid systems with easy gate tunability of the carrier concentration. To attain the AR effect in the QH regime at a graphene–S interface, we prepared high-mobility graphene by encapsulating a monolayer or bilayer graphene sheet between two thin and clean hexagonal boron nitride (hBN) crystal layers^16, 17^. Compared with the monolayer graphene (MLG), the bilayer graphene (BLG) had almost regular gap spacings between neighbouring Landau levels (Δ_LL,*n*_ = *E*_*n*+1_ − *E*_*n*_), except for the cases where *n* = 0, 1. Thus, BLG manifests more robust quantised QH conductance steps than in MLG, even for high filling factors. A good edge contact between a graphene layer and an Nb superconducting electrode with a high critical field (*H*_c2_ ~ 3.5 T) allows the superconducting proximity effect in the QH regime for a magnetic field above ~1 T.

To attain a stronger AR contribution, most of the previous experimental studies adopted Josephson junctions made of two N–S interfaces arranged sufficiently close to each other with an overlap of the superconducting order of the two electrodes^18–20^. However, a junction device with a short channel length allows only two-terminal measurements, where the voltage drop across the junction inevitably contains a mixture of longitudinal and transverse Hall voltages. Thus, in such a device, one cannot effectively separate the edge conductance from the bulk contribution. This even distorts the QH conductance plateaus, depending on the aspect ratio of the junction^21^. In sharp contrast to previous studies, in this study, we adopted a three-terminal measurement configuration, involving the QH edge states at both sides of a graphene–S interface to detect the longitudinal and Hall conductance separately. The edge channels exhibited conductance enhancement on both the upstream and the downstream sides of the superconducting electrode, providing convincing evidence for AR mediated by AES along the superconducting-proximity interface. The conductance enhancement in the QH regime was also confirmed by bias spectroscopy at a fixed magnetic field below the superconducting critical temperature, which manifested formation of the AES along the contact edge of the Nb superconducting electrode. In our measurements, the AR-induced conductance enhancement via the QH edge states was more evident in the QH plateaus as more channels participated in the Andreev process for high filling factors (ν ≥ 24). This indicates that each mode of edge channels participated in the AR process via the AES. In addition, the Fermi-energy-modulated background signals of the Landau-level gaps were observed in bias spectroscopy measurements, which were superposed on the AR signal near zero bias. Compared with Josephson junctions, our device configuration allows more precise determination of the relationship between the development of QH edge states and superconducting pair coherence. We find that a very recent work^22^ also used the same measurement configuration as ours. A three-terminal configuration was adopted in the study to confirm the crossed Andreev reflection in the QH states. Unlike the AES in this study, however, their result of the negative resistance in the downstream edge states, which represents the hole current, was caused by the nonlocal coherence between electrons and holes.

**Figure 1:**
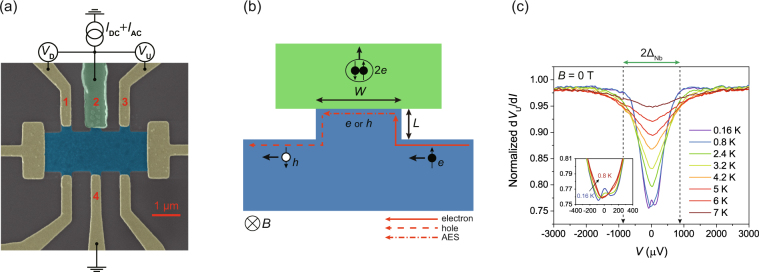
Graphene–superconductor hybrid device. (**a**) False-coloured scanning electron microscope image of the BLG device. (**b**) Formation of Andreev edge states (AES). Superconductor (green) and graphene layer (blue) are shown. The solid, dot-dashed, and dashed lines represent the incoming electron channel, AES, and outgoing hole channel, respectively. Incoming electrons and outgoing holes form a coherent paired state via the AES. The width of the interface and the length of the neck of the junction were *W* = 360 nm and *L* = 130 nm, respectively. (**c**) Bias spectroscopy of three-terminal differential resistance of the Nb contact for *B* = 0 and *V*_BG_ = 10 V. Here, d*V*_U_/d*I* at temperatures below *T*_c_ was normalised by d*V*_U_/d*I*_*T* = 8.4 K_. Inset shows a magnified view near zero bias for *T* = 0.16, 0.36, 0.6, and 0.8 K.

Figure 1(a) shows a false-coloured scanning electron microscope (SEM) image of the BLG hybrid device and the measurement configuration. The Nb superconducting electrode (green) is in contact with the BLG sheet (blue) between the two normal voltage probes (yellow) on the upper side of the BLG. A bias current *I* was applied through electrodes 2 and 4 (*I* = *I*_24_), while the upstream voltage *V*_U_ (=*V*_23_) and the downstream voltage *V*_D_ (=*V*_21_) were measured. In the QH regime in a high transverse magnetic field, carriers flowed in the chiral edge channels in the counter-clockwise direction, as shown in Fig. 1(b).

Carriers incident from the BLG to the superconducting contact form an AES, which consists of coherent paired states of Andreev-reflected electrons and holes flowing along the interface of the BLG–S junction. The AES maintains the coherence of hybridised electron-hole quasiparticles as long as the AR of quasiparticles occurs at the BLG–S interface. The coherence of the Andreev pairs is maintained both upstream and downstream of the AES within the range of pair breaking by the disorder-induced scatterings at the edges.

From the quasiclassical point of view, observation of the AR effect in the QH regime depends on the charge type of the outgoing quasiparticles at the exit point of the junction^8^. The proximity effect along the N–S interface becomes most evident when hole-like quasiparticles are dominantly injected downstream of the N–S interface into the edge conducting channel. In this approach, the charge type of outgoing carriers can be determined by the bouncing number (*N*) of quasiparticles at the interface and the probability of AR (*P*_AR_) upon bouncing. Thus, the dominance of either charge type of carriers varies depending on the width *W* of the N–S interface, magnetic field (*B*), and the interfacial barrier strength (*Z*).

To interpret the observed results in this study, we adopt the quantum mechanical scheme described in Ref. 23 for AR in a graphene–S system in the QH regime, considering the charge conversion probability between the normal edge state and the AES. In this scheme, unlike in 2DEG–S systems, the measured conductance of the QH plateaus in graphene depends on the valley polarisations of quasiparticles from the upstream and downstream edge states as $G=\,\frac{2{e}^{2}}{h}(1-\,\cos \,{\rm{\Theta }})$, where $\cos \,{\rm{\Theta }}(={{\boldsymbol{\nu }}}_{1}\cdot {{\boldsymbol{\nu }}}_{2})$ is the product of the valley isospins. These valley isospins are defined in the Bloch sphere for the upstream (${{\boldsymbol{\nu }}}_{1})\,\,$and downstream (${{\boldsymbol{\nu }}}_{2})$ flow along the opposite edge states, and ${\rm{\Theta }}$ is the angle between ${{\boldsymbol{\nu }}}_{1}$ and ${{\boldsymbol{\nu }}}_{2}$. The lowest QH plateau (*n* = 1 for MLG; *n* = 0 and 1 for BLG) depends most sensitively on the valley polarisations, since it can be strongly influenced by the types of edge termination. The zigzag edge has ${\boldsymbol{\nu }}=\pm \hat{{\boldsymbol{z}}}$ (from the three-dimensional unit vector in the Bloch sphere) depending on the graphene sublattice located at the edges. Thus, for a superconductor placed between opposite sides of zigzag edges, ${\rm{\Theta }}\,=\,{\rm{\pi }}$. The armchair edge has ${\boldsymbol{\nu }}\cdot \hat{{\boldsymbol{z}}}=\,0$ as the wave function does not exist in both sublattices, with the valley isospins lying on the *x-y* plane in the Bloch sphere. Because an Andreev pair of an incoming electron and an outgoing hole has opposite valley polarisation in graphene with^24^ $\cos \,{\rm{\Theta }}=-1$, the lowest QH conductance corresponding to *n* = 1 for MLG becomes $4\frac{{e}^{2}}{h}$, which is the same as for perfect Andreev reflection (*P*_AR_ = 1) in a system with time-reversal symmetry. In contrast, the states of *n* ≥ 2 for MLG are valley degenerate because they form further apart from the graphene edge than the *n* = 1 state, which leads to ${{\boldsymbol{\nu }}}_{1}\cdot {{\boldsymbol{\nu }}}_{2}\ne -1$. In this case, the Hall conductance of the *n* ≥ 2 states deviates from the doubled conductance for the perfect AR. At the same time, the intervalley scattering of quasiparticles during the propagation of edge channels reduces the AR probability. The effect of valley degeneracy for *n* ≥ 2 states and intervalley scattering at the graphene–S interface will be addressed in the discussion section. In addition to this valley-related consideration, Ref. 8 takes into account mode mixing in the edge channels near the corners of graphene–S interface. This is to describe the AR-induced conductance variation for *n* channels with the electron-hole conversion probability based on transfer matrices in the quantum mechanical treatment. In this scheme, one considers that the AR occurs for the inner edge channels for *n* ≥ 2 as well as the outermost edge channels.

Bias spectroscopy of the Nb superconducting contact was performed to confirm the contact transparency for *V*_BG_ = 10 V and *B* = 0 T. Figure 1(c) shows the temperature dependence of the upstream differential resistances normalised by the value taken at *T* = 8.4 K [=(d*V*_U_/d*I*)/(d*V*_U_/d*I*)_*T* = 8.4 K_] in the three-terminal configuration. Each curve shows a dip near zero bias, which suggests that AR occurred inside the superconducting energy gap of the Nb electrode. At the base temperature of 0.16 K, the zero-bias differential resistance drops by ~30%, indicating a highly transparent proximity contact at the graphene–Nb interface. The superconducting gap energy of Nb, Δ_Nb_, is estimated by choosing the bias voltage where the differential resistance starts dropping abruptly at *T* = 0.16 K [vertical dashed lines in Fig. 1(c)], where Δ_Nb_ is about 850 μV (V = Δ/*e*). The resistance dips are broadened and eventually disappear as *T* increases beyond the superconducting critical temperature of Nb (*T*_c_ = 8.1 K). Tiny zero-bias resistance peaks near the base temperature (*T* = 0.16 and 0.36 K) were usually found in our Nb-contacted devices (also found in MLG devices), which may suggest the presence of a small potential barrier at the interface; however, these resistance peaks appeared only at low temperatures (*T* < 0.6 K). Thus, we suggest that they were caused by the reentrance effect with the high transparency between the superconductor and the neighbouring normally conducting (Au) electrodes that act as thermal reservoirs^25, 26^.

## Results

### Andreev reflection via quantum-Hall edge states

**Figure 2:**
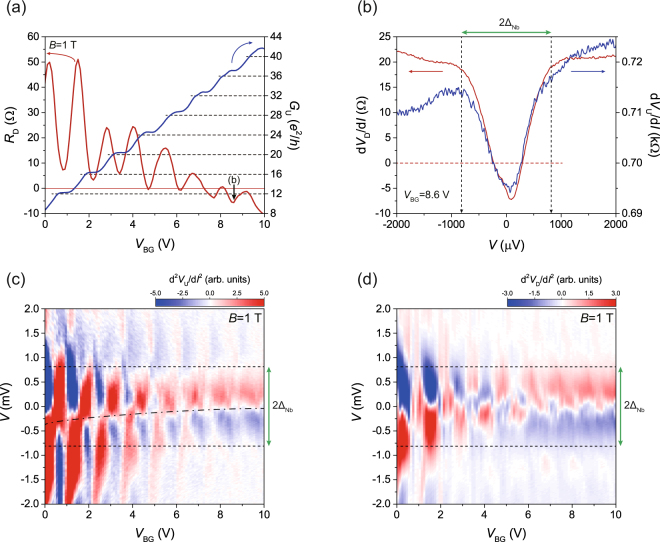
Andreev reflection via quantum-Hall (QH) edge states. (**a**) Back-gate-voltage dependence of zero-bias resistance of the downstream edge states (*R*_D_, red line) and zero-bias conductance of the upstream edge states (*G*_U_, blue line). The horizontal broken lines represent the standard quantised QH conductance. The red line is located on zero resistance of *R*_D_. The black arrow shows the back-gate voltage location where the data of Fig. 2(b) were measured. (**b**) Bias dependence of the upstream (red curve) and downstream (blue curve) differential resistance for edge states at the back-gate voltage of 8.6 V. The two curves have similar dip structures with the gap size almost equal to Δ_Nb_. (**c,d**) Colour maps for the bias dependence of the curves d^2^ *V*_U_/d*I* ^2^ and d^2^ *V*_D_/d*I* ^2^ as a function of back-gate voltages, respectively. Dashed lines show the voltage scales of *V* corresponding to 2Δ_Nb_.

**Figure 3:**
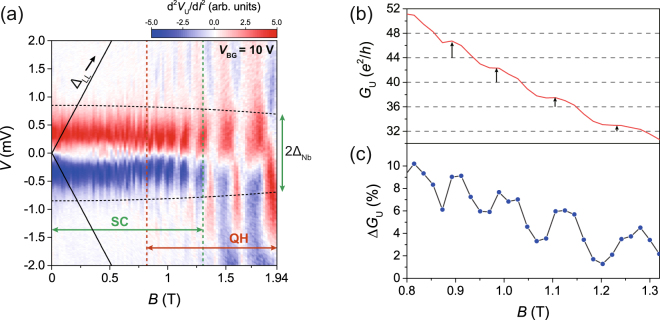
*B*-field dependence of conductance enhancement. (**a**) A colour map for the bias dependence of d^2^ *V*_U_/d*I* ^2^ with varying *B* field for *V*_BG_ = 10 V. The solid black lines represent the gap energies of Landau levels, Δ_LL_. The vertical broken lines show the boundary between the superconducting proximity effect and the quantum-Hall effect regions. (**b**) The line cut at zero bias as a function of *B*-field, obtained from Fig. 3(a) (red line). (**c**) The blue-dot–solid-line plot represents the conductance enhancement Δ*G*_U_ estimated from *G*_U_.

Figure 2(a) shows the back-gate voltage (*V*_BG_) dependence of the downstream resistance *R*_D_ (=*V*_D_/*I*) and the upstream conductance *G*_U_ (=*I*/*V*_U_) measured at *B* = 1 T. Considering the contribution from AR, it can be noted that *V*_U_ = *V*_23_ = *e*(μ′_2_ − μ_3_) = *e*(μ_2_ − μ_3 + _μ_AR_ + μ_c_) = *e*(μ_Hall_ + μ_AR_ + μ_c_) and *V*_D_ = *V*_21_ = *e*(μ′_2_ − μ_1_) = *e*(μ_2_ − μ_1_ + μ_AR_ + μ_c_) = *e*(μ_AR_ + μ_c_), respectively. Here, the chemical potential of probe 2 is μ′_2_ = μ_2_ + μ_AR_ + μ_c_, where μ_AR_ and μ_c_ are the chemical potentials that arise from the AR and the contact resistance of the Nb junction. μ_Hall_ [ = μ_1_ − μ_3_] is the Hall potential drop and μ_1_ = μ_2_. μ_AR_ gives a negative voltage drop (μ_AR_ < 0) to both *V*_U_ and *V*_D_ when AR occurs. *R*_D_ shows the development of Landau levels, with minima when the Fermi level of graphene is between the neighbouring Landau levels. *R*_D_ does not vanish completely at these incompressible states until *V*_BG_ reaches 4.7 V, which corresponds to a filling factor ν = 24. The residual resistance of a few ohms at each minimum for ν < 24 may result from backscattering in the edge channels and the contact resistance at the graphene–S interface. *G*_U_ shows the quantised conductance arising from the unique Landau level structure of BLG [σ_xy_ =  ± (4*e* ^2^/*h*)∙*n* for *n* ≥ 1, where *n* is integer]^27^. For high filling factors, the conductance plateaus are clearly enhanced from the expected values denoted by dashed lines, with stronger conductance deviation as more edge states participated in the AR (details are discussed below in relation to Fig. 3). Above *V*_BG_ = 7.3 V, *R*_D_ becomes negative. Even in the compressible state between the plateaus of *G*_U_ at *V*_BG_ = 9.2 V, a large number of edge channels participate in AR, resulting in negative downstream resistance.

For better clarification, we performed bias spectroscopy for both *V*_D_ and *V*_U_ simultaneously as a function of *V*_BG_ for *B* = 1 T. Figure 2(b) shows the bias dependence of both the upstream and the downstream differential resistance at a filling factor of ν = 36 (*V*_BG_ = 8.6 V). The vertical dashed lines (black) indicate the value of Δ_Nb_ (~813 μ*e*V) for *B* = 1 T, calculated using Bardeen-Cooper-Schrieffer (BCS) theory with the measured zero-field gap energy of Nb. The measured energy range of the dip structure in the upstream resistance (blue) agrees well with the calculated Δ_Nb_, providing additional confirmation that it indeed arises from the superconducting proximity effect. This structure was also found in the downstream resistance (red) with almost the same energy scale as Δ_Nb_, which indicates that incident electrons from the upstream side were paired coherently with outgoing holes via the AES.

To examine clearly the progressive evolution of the superconducting proximity effect on the edge states for different filling factors, we subtracted the background in the differential resistance by obtaining d^2^ *V*/d*I* ^2^, the second derivative of *V*_U_ or *V*_D_ with respect to the bias current *I*, as shown in the colour maps in Fig. 2(c) and (d). The red (blue) colour in the maps represents an increase (decrease) in the differential resistance with a positive increase in bias current. For ν < 24 (or *V*_BG_ < 4.7 V), the alternating peak and dip structures near zero bias appeared with modulating *V*_BG_, caused by Landau levels with inter-level gap energies exceeding the value of Δ_Nb_ at *B* = 1 T. A recent report on bias spectroscopy in suspended BLG in the QH regime reveals that these features arise as the Fermi level passes through the different Landau levels with increasing bias^28^. Beyond the filling factor of ν = 24 (or *V*_BG_ > 4.7 V), however, these features are gradually replaced by successive zero-bias dips in the differential resistance due to the superconducting proximity effect. Horizontal dashed lines represent the energy scale of 2Δ_Nb_. The centres of peak or dip structures of the differential resistance (dot-dashed line) deviate from zero bias at low filling factors for *V*_BG_ < 4.7 V but return gradually to zero bias at high filling factors. As mentioned above, increasing the bias current enables the bulk conduction to contribute to the transport. Then, the differential resistance can become asymmetric depending on the polarity of the bias because, in this case, the current flows through different paths, which causes the alternating peak-dip centres to deviate from the zero bias for *V*_BG_ < 4.7 V. However, as the AR is not affected by the bias polarity, the centres of dip structures are located near zero bias in the region of the superconducting proximity effect for *V*_BG_ > 4.7 V.

The colour map of d^2^ *V*_D_/d*I* ^2^ shown in Fig. 2(d) also presents similar results to the ones in Fig. 2(c). Alternating peak-dip structures of differential resistance also appear for ν < 24. The variation of d*V*_D_/d*I* near the incompressible states (corresponding to the dips of *R*_D_) is smaller than in the compressible states between the adjacent Landau levels (corresponding to the peaks of *R*_D_). This is because *V*_D_ drops by a few V (*R*_D_ < 10 Ω) near the incompressible states, which makes the bias dependence weaker than in the compressible states. Increasing the Fermi level, the superconducting proximity effect was more evident in both the upstream and the downstream edge states for ν > 24. This indicates that the same potential deviation μ_AR_ due to the AES was detected in both *V*_U_ and *V*_D_.

### Magnetic field dependence of conductance enhancement

Figure 3(a) shows a two-dimensional colour map of d^2^ *V*_U_/d*I* ^2^ as a function of *B* field and the bias voltage *V* for *V*_BG_ = 10 V. The near-horizontal dashed curves (black) represent the energy scale of 2Δ_Nb_ calculated by the BCS theory. The *B*-field ranges denoted by the green and red arrows represent the regions where the superconductivity survived with the AR effect and the clear QH plateau developed, respectively. It clearly exhibits the *B*-field region, 0.8 T ≤ *B* ≤ 1.32 T, where the superconducting proximity and the QH effect coexist. Within this range, one clearly observes both the AR-induced resistance dips for *V* < | Δ_Nb_/*e*| and the alternating background of the differential resistance due to the QH effect for *V* > | Δ_Nb_/*e*|. For *B* > 1.32 T, the dip structure from the AR is no longer present. Only the alternating peak-dip structures are visible with increasing *B* in the energy range beyond 2Δ_Nb_. The solid black lines in Fig. 3(a) show the calculated gap energy of Landau levels^28^ (Δ_LL_) for a given *B*, which is much larger than 2Δ_Nb_ in the coexistence zone. It indicates that Δ_LL_ is irrelevant to the conductance behaviour appearing for *V* < | Δ_Nb_/*e*| in Figs 2 and 3.

Focusing on the coexistence of the two effects in Fig. 3(b), one notes that the zero-bias differential conductance *G*_U_(*B*) (red) is enhanced above the expected value of 4e^2^/*h* ∙ *n* for the *n*-th conductance plateau. This is caused by the AES, which is present in all of the plateaus in the coexistence zone in Fig. 3(b). The conductance enhancement is estimated in Fig. 3(c) as Δ*G*_U_ = (*G*_U,AR_ − *G*_U,N_)/*G*_U,N_ (blue dots), where *G*_U,AR_ is the maximum conductance enhancement by AR and *G*_U,N_ is the normal upstream conductance for *V* > |Δ_Nb_/*e*|. Δ*G*_U_ tends to decrease monotonically along with decreasing *G*_U_ as the participating edge channels are reduced with increasing *B* field. Δ*G*_U_ shows oscillating behaviour, the maxima (minima) of which correspond to treads (risers) of the quantised steps in *G*_U_. In Fig. 2(c), the bias-spectroscopy measurements in the QH regime reveal a peak-dip structure with the same periodic behaviour as the QH plateaus. Because the treads and risers of the QH plateau steps are related to the peaks and dips in the differential resistance, respectively, the oscillating characteristic of Δ*G*_U_ arises from the AR signals that were superimposed positively or negatively by the modulated Landau-level background signals at zero bias. In addition to the quantised conductance of *G*_U_, this behaviour reconfirms the evidence for QH edge states in the AR-QH coexistence zone.

### Temperature dependence of Andreev edge states

**Figure 4:**
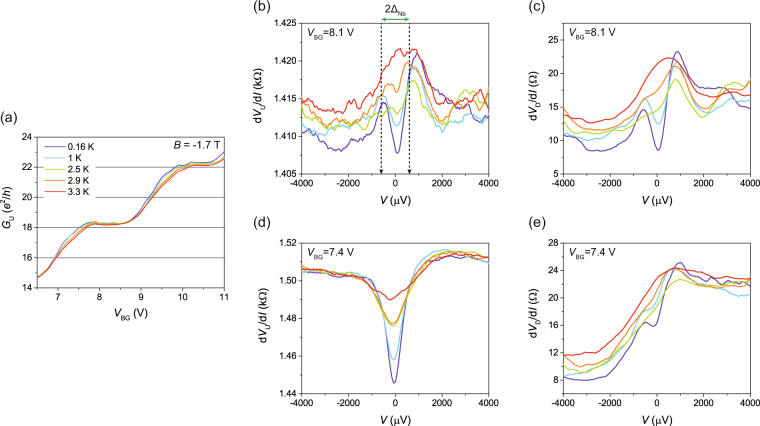
Temperature dependence of Andreev edge states in monolayer graphene. (**a**) A set of quantised conductances for the upstream edge states with varying temperatures in a monolayer graphene device. (**b**,**c**) Bias dependences of d*V*_U_/d*I* and d*V*_D_/d*I* in the incompressible states for *V*_BG_ = 8.1 V, respectively. (**d**,**e**) Bias dependences of d*V*_U_/d*I* and d*V*_D_/d*I* in the compressible states for V_BG_ = 7.4 V, respectively. For (**a**–**e**), line plots have similar profiles for *T* = 0.16, 1, 2.5, 2.9, and 3.3 K, with the same colour codes as in (**a**).

We also confirmed the existence of AES in an MLG device at variable temperature up to the critical temperature of Nb, *T*_c_ = 3.3 K, for *B* = −1.7 T. We found that a BLG device was not suitable for such measurements because BLG shows more thermal broadening of the Landau levels than MLG at a given temperature and a resultant smearing of the QH plateaus (see the supplementary material for the *T* dependence of the BLG device). Similar device and measurement configurations were adopted for measurements using the MLG device, except for a polarity change of the applied magnetic field (*B* = −1.7 T). With the opposite polarity of magnetic field, the locations of the voltage probes *V*_U_ and *V*_D_ were also switched accordingly. The MLG device gave similar results to the ones obtained using the BLG device. The MLG–Nb junction device revealed a zero-bias conductance enhancement of ~14% with Δ_Nb_ ~570 μV for *B* = 0 T and *T* = 0.16 K. Figure 4(a) shows a set of line plots of *G*_U_ at the QH plateaus of ν = 18 and 22 in the MLG device for *T* = 0.16, 1, 2.5, 2.9, and 3.3 K. Each QH plateau gradually recovers the normally quantised value as temperature rises. The temperature dependence occurs across the entire *V*_BG_ range in Fig. 4(a) for both incompressible and compressible states of the QH regime; however, the compressible states seem to have a larger temperature dependence. We believe that, in addition to the edge channel conduction, the bulk channels in the compressible states also contributed to the AR.

Figure 4(b–e) show the corresponding d*V*_U_/d*I* (b, d) and d*V*_D_/d*I* (c, e) as a function of bias voltage *V* with the same colour conventions as in Fig. 4(a) for both the upstream and the downstream edge states. Here, Fig. 4(b) and (c) are the results near the incompressible state (*V*_BG_ = 8.1 V), so that the development of the zero-bias resistance dips is clearly visible for *V* < |Δ_Nb_/*e*| with almost the same background shape of d*V*/d*I* for both the upstream and the downstream edge states. In this device, the incoming electrons from the upstream edge states and the outgoing quasiparticles to the downstream ones are coherently coupled by the AES along the interface of the junction for *V* < |Δ_Nb_/*e*|. As backscattering is almost ruled out in the incompressible states, both *V*_U_ and *V*_D_ measure the μ_AR_ induced via the AES. On the other hand, Fig. 4(d) and (e) are the results close to the compressible states (*V*_BG_ = 7.4 V), where the backscattering in the bulk transport channels also contributed to the transport. The bulk transport channels of the MLG contained randomly distributed defects, which should have acted as scattering sources for the quasiparticles in the AES, resulting in decoherence between quasiparticles. Consequently, as seen in Fig. 4(d–e), the observed d*V*_U_/d*I* and d*V*_D_/d*I* exhibit a different bias-dependent background. Their asymmetrical background and the much weaker superconducting proximity effect in the downstream edge state result from the backscattering and decoherence in the AES. We argue that the similarity in the *I*–*V* characteristics between the upstream and downstream edge states is the important criterion for distinguishing superconducting proximity effects via QH edge states without backscattering.

## Discussion

Non-ideal transparency of the graphene–S interface (Δ*G*_U_ ~30% at *B* = 0 T in BLG) and valley-degenerate edge states (*n* ≥ 2) partly breaks the Andreev pairs in the two opposite edge states, which reduces the enhancement of QH conductance from the expected doubled QH conductance (case for *P*_AR_ = 1). These additional factors causing a deviation from the perfect AR are hard to define quantitatively because of the presence of very subtle and complicated factors such as inhomogeneous transparency at the junction interface. Thus, we introduce a simple AR conversion factor α to the Hall conductance equation from ref. 23, $G=\,\frac{2{e}^{2}}{h}(1-{\rm{\alpha }}\,\cos \,{\rm{\Theta }})$. Setting ${\rm{\Theta }}={\rm{\pi }}$ for the case where all incoming electrons are converted into outgoing holes with opposite valley polarisation, the factor α acts as the portion of the outgoing hole-like quasiparticles that are coherently coupled with incoming electrons via the AES. The value of this AR conversion factor α, corresponding to the accumulated conductance enhancement for the channels up to *n* = 10 level, is estimated to be ~0.057 from Δ*G*_U_ taken at zero bias.

In the *B*-field dependence of the AR effect of Fig. 3, no sign of conductance oscillation is seen as the *B* field varies within the QH regime. In the quasiclassical treatment, the outgoing quasiparticles can be either electron-like or hole-like alternatively as the bouncing number of quasiparticles at the 2DEG–S interface is varied with *B* field^9^. This conductance oscillation is treated quantum mechanically in terms of the phase factor acquired by the quasiparticles along the AES of the 2DEG–S interface. However, the unique valley isospin degrees of freedom in graphene of a graphene–S junction makes the electron-hole mixed states degenerate, which leads to vanishing of the phase factor to the quasiparticles in the AES^8^. Therefore, the conductance is mainly determined by the electron-hole conversion probability factor α via the AES. Although the control of this probability in measurements remains uncertain, we observed only the conductance enhancement in three different graphene devices used in this study.

While conductance enhancement by AR was clearly identified at higher filling factors (*n* ≥ 6), the superconducting proximity effect was barely observed for lower filling factors of the QH edge states in both the BLG and the MLG devices (see supplementary materials for the MLG device). This feature is contrasted with the result from graphene Josephson junctions in other works^18–20^ where the AR effect was observed for low filling factors also. Surely the outermost edge states of *n* = 0, 1 have a unique dependence on the valley polarisations for incoming and outgoing quasiparticles, and can be smeared easily due to the strong intervalley scattering by edge disorders. However, it does not explain why the other QH edge states from the lower filling factors (1 < *n* < 6) in our study did not exhibit the superconducting proximity effect via the AES. In an S–N–S proximity Josephson junction, with the normal-conducting channel as a weakly superconductive link, the coherence of Andreev pairs can be maintained much stronger than in a single N–S junction. The strong proximity effect in a Josephson junction allows a conductance enhancement even for low filling factors with edge channels fewer than in a single N–S junction for a given temperature and *B* field. Therefore, the relatively weak strength of the proximity effect in our N–S junction device led to the observation of the conductance enhancement only when sufficiently large number of QH edge channels participated in the AR process along the AES for high filling factors.

Moreover, intervalley scattering can break the coherence between the incoming electrons and the outgoing quasiparticles via the AES for smaller values of ν. The width of the junction interface *W* plays an important role in quasiparticle coherence in our devices, as it determines the propagation lengths of each AES. Because the smaller-ν edge states are located on the outer of the conducting channels, an AES of a lower filling factor requires a greater coherence length than that of a higher filling factor. The localisation length of an edge channel in the QH regime is represented by the cyclotron radius ${r}_{c}\,=\,\frac{\hslash {k}_{{\rm{F}}}}{eB}$, where $\hslash$ is the reduced Planck constant, ${k}_{{\rm{F}}}$ is the Fermi wavenumber and *e* is the elementary charge. With ${k}_{F}=\,\sqrt{\pi {n}_{{\rm{g}}}}$, where ${n}_{{\rm{g}}}$ is the carrier concentration for MLG or BLG, the QH edge channels are confined within ${r}_{c}\,=\,{l}_{B}\sqrt{\nu /2}$. Therefore, the propagation length of AES (*l*_AES_) can be determined by the strength of the magnetic field (*B*) and the width of the superconducting interface (*W*). The BLG device with *W* = 360 nm, shown in Fig. 1(a), started to show the superconducting proximity effect from ν = 24 (*n* = 6), which corresponds to *l*_AES_ ~187 nm with *l*_B_ ~25 nm (*B* = 1 T). In a control experiment with an MLG device with a shorter superconducting contact edge (*W* = 270 nm), the superconducting proximity effect began to appear at a lower filling factor, ν = 18 (*n* = 5). Here, *l*_AES_ ~120 nm for the same *B* field and *T* (see supplementary materials for the MLG experiment). It is proposed in ref. 23 that the Hall conductance with intervalley scattering at the graphene–S interface is $G=\,\frac{2{e}^{2}}{h}(1-{e}^{-\frac{{\rm{\Gamma }}W}{{v}_{0}}}\,\cos \,{\rm{\Theta }})$, where Γ is the intervalley relaxation rate and *v*_0_ is the velocity at the junction interface. These results seem to support the above argument concerning the coherence of the AES. One cannot reduce *W* too much to obtain the proximity effect at lower filling factors because a narrow contact edge often results in an effective channel disorder, which backscatters the incoming electrons from the QH edge channels and results in suppressed QH conductance^29^.

In conclusion, we fabricated ballistic MLG-Nb and BLG-Nb hybrid devices and observed the AR effect via the QH edge states that form in a strong transverse magnetic field below the superconducting critical field and critical temperature of an Nb electrode. In contrast to the two-terminal measurements in previous studies, the three-terminal measurement configuration adopted in this study allowed us to obtain detailed evidence for AR mediated by AES excluding the bulk contribution. From the observed negative resistance on the downstream side of the AES, coherent conversion of electrons from the upstream side of the AES into paired holes was clearly confirmed. The AR-induced conductance enhancement was more evident as more edge modes participated in the AR process, as the AR conversion probabilities depend on valley degeneracy and intervalley scattering. This study provides valuable detailed information on the propagation of superconducting coherence with specific chirality along edge channels in the QH regime. It also provides a new scheme for investigating the interplay between superconductivity and the chiral edge conducting state that often emerges in 2D topological materials.

## Method

### Device fabrication

Our MLG and BLG hybrid devices were fabricated in the following way. First, we encapsulated graphene between 20–30-nm-thick hBN crystals using the sequential stamping method^17^. Graphene was protected from ambient conditions by the hBN layers and the polymer residue that could not be completely removed after the lithography processes. The encapsulation enhanced the mean free path of the graphene significantly beyond the size of the graphene layers in our devices. Then, the encapsulated graphene was placed onto a heavily electron-doped silicon substrate with a 280-nm-thick SiO_2_ capping layer, which was used to apply the back-gate voltage (for both MLG and BLG devices). After choosing a defect-free surface on the encapsulated graphene layer under atomic force microscopy, standard electron beam lithography and successive plasma-etching were adopted to prepare the edge contact for the metallic electrodes. A bilayer electrode of 10-nm-thick Cr and 60-nm-thick Au layers was deposited by standard electron-gun evaporation. A 100-nm-thick Nb electrode was deposited by DC magnetron sputtering after inserting a 10-nm-thick Ti buffer layer by electron-gun evaporation between the graphene and Nb. The Ti buffer layer enhanced the adhesion of the electrode and improved the contact characteristics, and thus prevented damage to the graphene layer during the sputtering process.

### Measurements

All devices were mounted on a dilution fridge system (Kelvinox, Oxford Instruments) with a base temperature of 150 mK. Electrical connection of the measurement probe lines of the fridge system was made via two stages of low-pass RC filters. All measurements data were obtained using a current-biased standard lock-in amplifier technique operated at a frequency of 17.77 Hz. *I*–*V* characteristics of the device were measured using DC current biasing mixed with an AC bias current of 100 nA.

### Estimation of the conductance enhancement

In relation to Fig. 3(b), *G*_U,AR_ was determined by taking the maximum conductance near zero bias. Heavy data averaging was adopted to obtain the differential conductance *G*_N_ in the bias range *V* > |Δ_Nb_/*e*| to reduce the irregular background differential conductance caused by the QH edge channels.

## Electronic supplementary material

Supplementary Information
