# Supplementary material for: Propagation of superconducting coherence via chiral quantum-Hall edge channels
Source: Sci Rep. 2017 Sep 8;7:10953. doi: 10.1038/s41598-017-11209-w (PMC5591196; doi:10.1038/s41598-017-11209-w)
Supplement: Supplementary file 1 — Supplementary Information [file 41598_2017_11209_MOESM1_ESM.pdf]

Supplementary Information

Propagation of superconducting coherence via chiral quantum-Hall edge channels

Geon-Hyoung Park[1], Minsoo Kim[1], Kenji Watanabe[2], Takashi Taniguchi[2], and Hu-Jong Lee[1*]

[1]*Department of Physics, Pohang University of Science and Technology, Pohang 790-784, Republic of Korea.*
[2]*Advanced Materials Laboratory, National Institute for Materials Science, 1-1 Namiki, Tsukuba 305-0044, Japan.*

[*]Correspondence and requests for materials should be addressed to H.-J.L. (email: hjlee@postech.ac.kr)

1. Device information

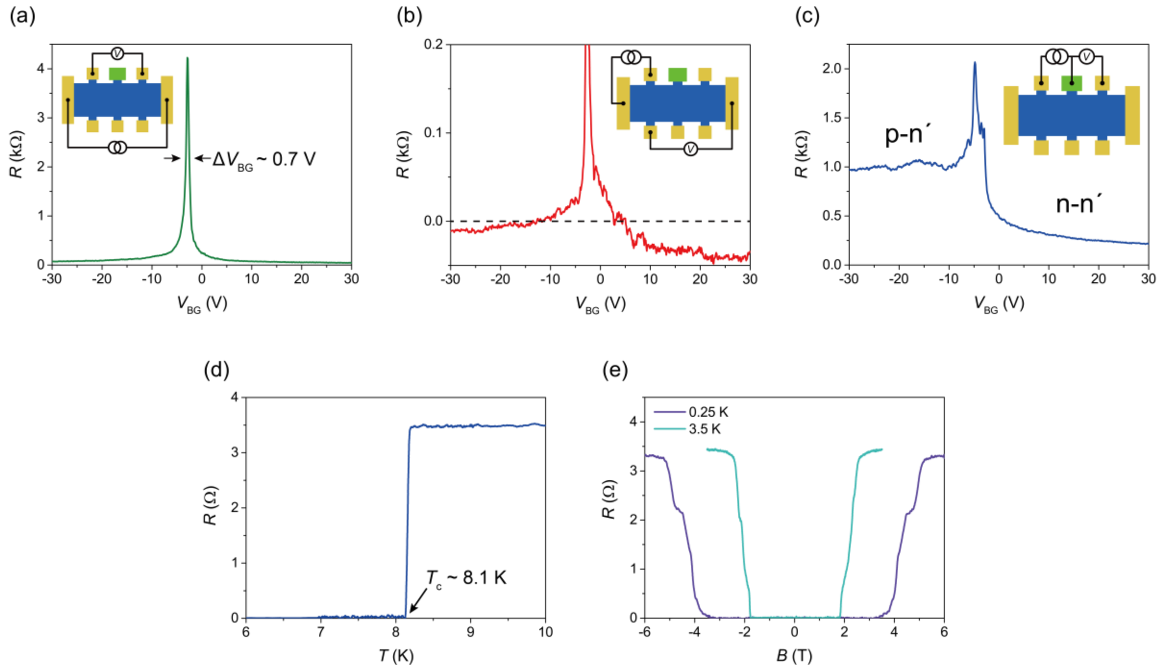

Figure S1 | Bilayer graphene (BLG) hybrid device. (a) Back-gate-voltage dependence of the resistance of the BLG layer at $T = 0.16$ K. ΔV_{BG} represents the full-width-at-half-maximum (FWHM) of the charge-neutral peak. **(b)** Resistance of the BLG layer in the Van der Pauw-like measurement configuration, showing the ballistic transport characteristics at $T = 0.16$ K. **(c)** Contact resistance of the superconductor-BLG junction at $T = 0.16$ K. Insets in (a-c) show each measurement configuration in the figures. **(d)**, **(e)** The critical temperature T_c and the upper critical field H_{c2} of the Nb electrode.

Before entering the quantum Hall (QH) regime, we examined the general electrical transport properties of bilayer graphene (BLG) and the superconducting transition of the Nb

electrode. Figure S1(a) shows results of conventional four-terminal voltage measurement as a function of back-gate voltage (V_{BG}) at T = 0.16 K. A pronounced and sharp charge neutrality point (CNP) of BLG is located at $V_{BG} = -2.9$ V, which means that our BLG is slightly electron-doped. We can roughly estimate the fluctuation level of the carrier density, Δn_g from the full-width-at-half-maximum (FWHM, ΔV_{BG}) of the CNP. Considering that the substrates for the BLG was overlaid with a 280 nm-thick SiO_2 layer and a 20 nm-thick hexagonal boron nitride (hBN) crystal, $\Delta V_{BG} \sim 0.7$ V can be converted into $\Delta n_g \sim 5 \times 10^{10}$ cm^{-2}, representing carrier density disorder smaller than that of graphene layers on bare SiO_2. Since the diffusive Drude model for charge carrier mobility ($\mu = \Delta\sigma/n_g e$) cannot be applied to the quasi-ballistic transport, we adopted another four-terminal measurement configuration, the Van der Pauw-like method, as the inset in Fig. S1(b)[1]. In this configuration, ballistically transporting electrons can reach the voltage probe located in the direction of the transport (the usual higher potential voltage probe) first. It gives a negative resistance except the very close to the CNP, where the n_g is extremely small. Observation of a negative resistance means that the mean-free-path of the BLG sheet exceeds the physical size of the device. Figure S1(c) shows the back-gate-voltage dependence of the Nb contact at T = 0.16 K. The contact resistance has asymmetrical bipolar behaviour because the Ti/Nb bilayer contact to graphene usually electron dopes the graphene layer and forms a p-n potential barrier[2]. Therefore, we focused on the electron doped region ($V_{BG} > -2.9$ V) of the BLG layer to enhance the Andreev reflection (AR) probability by avoiding the p-n potential barrier. The critical temperature ($T_c \sim 8.1$ K) and the upper critical field ($H_{c2} \sim 3.5$ and 1.8 T at T = 0.25 and 3.3 K, respectively) of the Nb electrode were also measured [Fig. S1(d), (e)].

2. Additional bias spectroscopy data in the BLG device (B = 1 T)

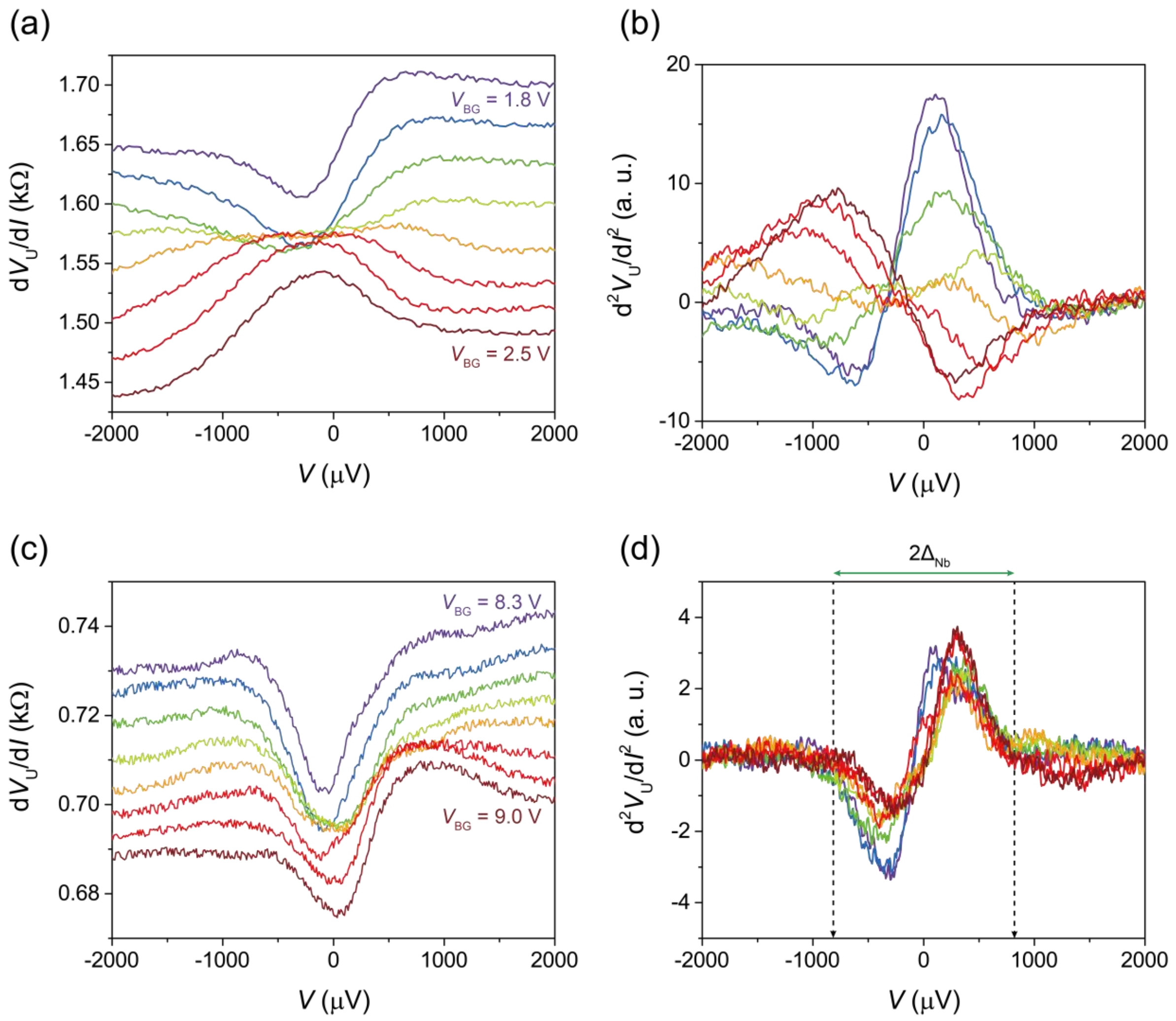

Figure S2 | Additional bias spectroscopy data in bilayer graphene device. (a) dV_U/dI for the back-gate voltage from 1.8 to 2.5 V in intervals of 0.1 V. **(b)** d^2V_U/dI^2 in the same range of bias voltages. **(c)** dV_U/dI for the back-gate voltage from 8.3 to 9.0 V in intervals of 0.1 V. **(d)** d^2V_U/dI^2 in the same range of bias voltages.

Figure S2(a) shows a set of bias dependence of dV_U/dI corresponding to Fig. 2(c). The curves for V_{BG} = 1.8 − 2.5 V represent the Coulomb-diamond-like bias dependence of the differential resistance arising from the presence of Landau level gap. These peak and dip structures near zero bias are merged at a specific resistance which corresponds to the QH plateau of ν = 16. The gap energy of the Landau level, Δ_{LL}, can be estimated from the half-width of the peak and dip curves at the QH plateau. The ideal value of $\Delta_{LL,\nu=16}$ ~ 3.8 meV can be calculated by the equation of the Landau level energy for bilayer graphene, $E_n = \frac{\hbar eB}{m^*}\sqrt{n(n-1)}$, where $\hbar$ is Planck's constant, e is the elementary charge, m^* ~ 0.02 – 0.04m_e is the effective mass of electron, and m_e is the rest mass of electron[3]. But Δ_{LL} can be smeared by the degradation of the graphene layer and thermal broadening effect. To remove background, the second derivative of Fig. S2(a) are performed as shown in Fig. S2(b) with the same colour code. The alternating sine-like curves near zero bias correspond to the peak and dip of the curves in Fig. S2(a). A rough estimation leads to Δ_{LL} > 3 meV. In contrast, the resistance dip structures from the AR become dominant at high filling factors [Fig. S2(c)]. Figure S2(d) showing consistent dip-peak curves of d^2V_U/dI^2 (*i.e.*, the resistance dip) at zero bias. The corresponding colour map of d^2V_U/dI^2 for the full range of back-gate voltages is presented in the main text [Fig. 2(c)].

3. Temperature dependence of Andreev edge states in bilayer graphene device (B = 1 T)

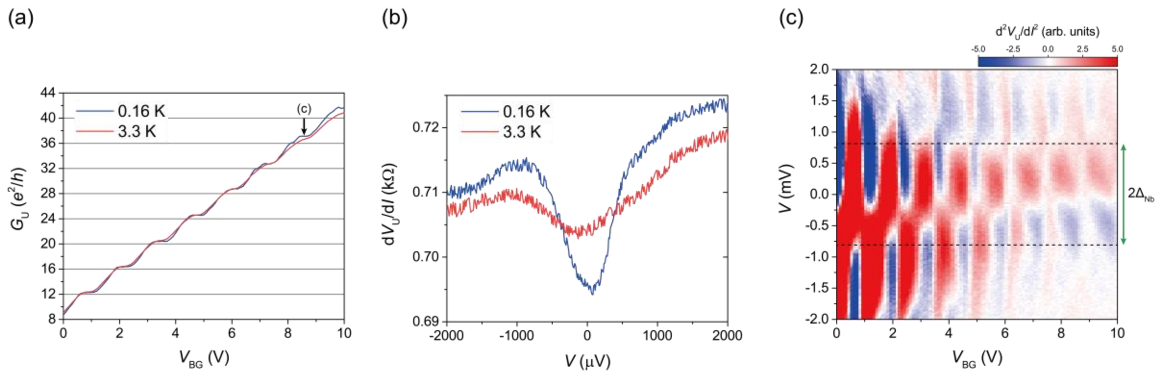

Figure S3 | Temperature dependence of conductance on the upstream side of edge states. **(a)** Comparison of G_U at $T = 0.16$ K (blue) and $T = 3.3$ K (red). **(b)** Bias dependence of dV_U/dI at $V_{BG} = 8.6$ V. The blue and red curves were obtained at $T = 0.16$ K and 3.3 K, respectively. **(c)** The colour map of d^2V_U/dI^2 as a function of the back-gate voltage at $T = 3.3$ K.

Since the distribution of localised states in the Landau levels in BLG was thermally broadened[4], the superconductivity of Nb electrode was not completely suppressed with well-developed QH plateaus at $B = 1$ T. At $T = 3.3$ K, we measured a set of I-V characteristics from at the upstream QH edge states, V_U. Figure S3(a) shows that QH plateaus are still robust at $T = 3.3$ K (red). Note that, comparing to the results at $T = 0.16$ K (blue), the conductance enhancement from the AR at $T = 3.3$ K is rather suppressed in high filling factors ($\nu \geq 36$). The bias spectroscopy of dV_U/dI at $V_{BG} = 8.6$ V is shown in Fig. S3(b). The AR signal at zero-bias at $T = 3.3$ K (red) decreased by half comparing with the data at $T = 0.16$ K. Normal resistance ($|V| > e\Delta_{Nb}$) is slightly decreased since the broadened localised states may have

contributed to a residual conductance to V_U. Fig. S3(c) shows the colour map of d^2V_U/dI^2 (second derivative of dV_U/dI) with varying V_{BG} at $T = 3.3$ K. In the same colour scale, we reconfirmed that the resistance dip structure ($\nu \geq 36$) was a little weakened by increasing temperature.

4. Additional *B*-field dependence of *I*-*V* characteristics in the bilayer graphene device

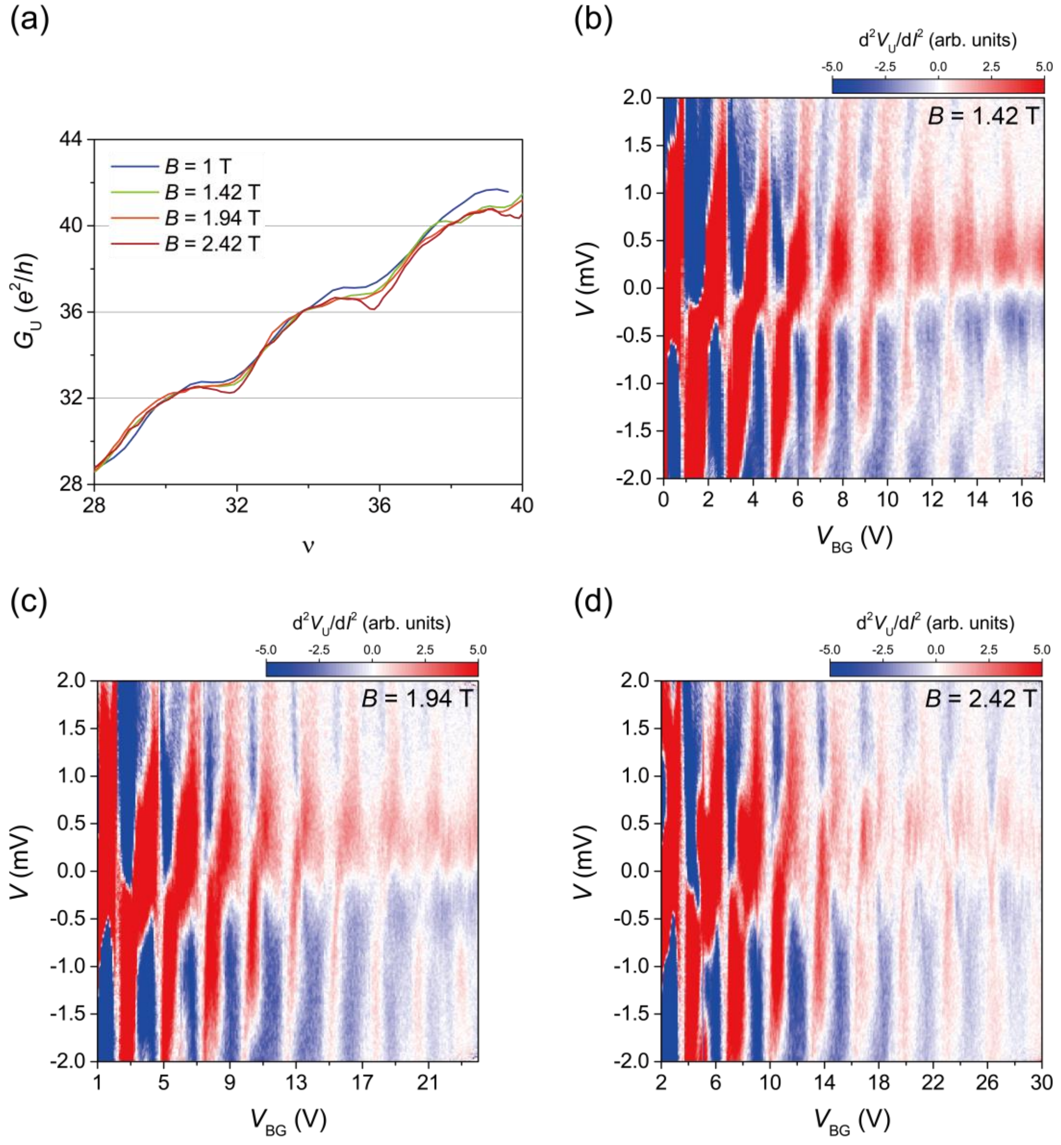

Figure S4 | Additional results of bias spectroscopy from V_U with increasing B-field. (a) A series of plots of quantised conductance from G_U as a function of filling factor ν. **(b-d)** The colour maps for the bias spectroscopy results of d^2V_U/dI^2 with varying V_{BG} at B = 1.42, 1.94, and 2.42 T, respectively.

A set of conductance lines from the upstream edge states as a function of filling factor $\nu = n_g h/eB$ with increasing B-field is shown in Fig. S4(a), where h is the Planck constant. We also took the bias spectroscopy as a function of V_{BG} as shown in Figs. S4(b)-(e). As the B-field gets stronger, the conductance enhancement gradually disappears at the QH plateaus, which occurs concurrently as the AR-induced resistance dip is replaced by alternating peak-dip structures arising from the Landau-level gap in the colour maps[3].

5. Additional *I-V* characteristics in monolayer graphene (MLG) device (T = 0.16 K, B = 1 & –1.7 T)

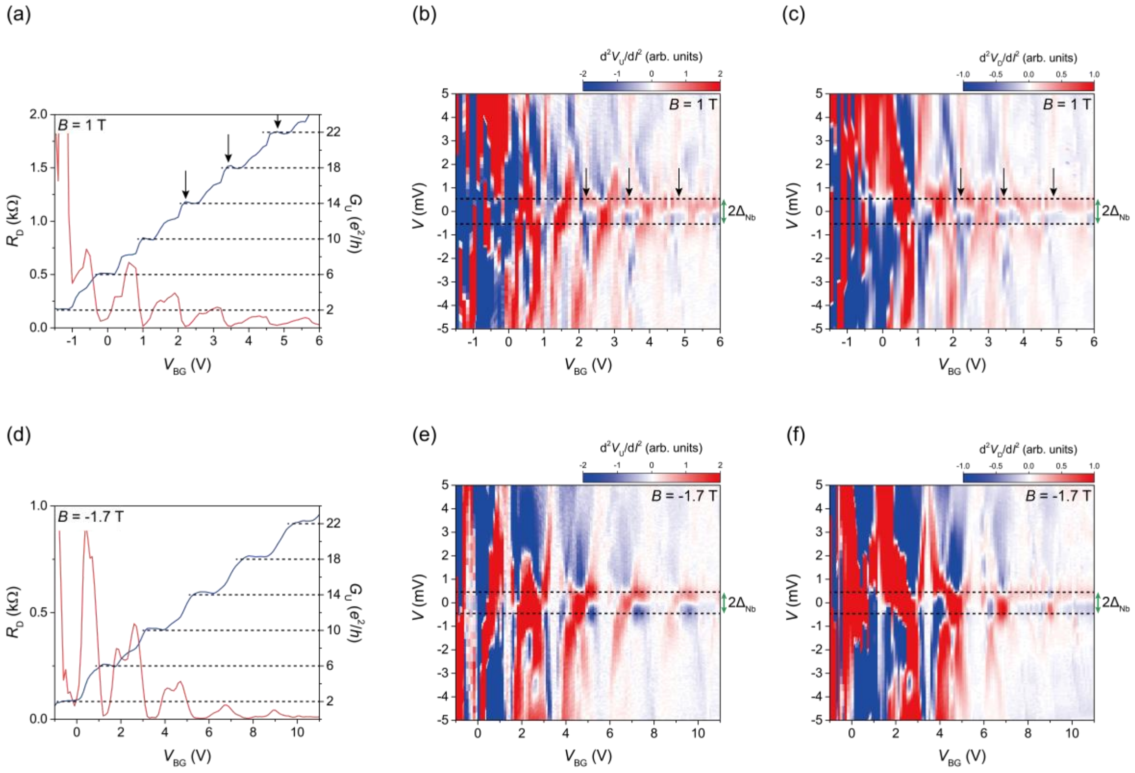

Figure S5 | Andreev reflection via quantum Hall (QH) edge states in the monolayer graphene device. (a) Zero-bias resistance (red) and conductance (blue) from V_D and V_U at B = 1 T. Dashed lines show the standard QH conductance. **(b), (c)** A set of d^2V/dI^2 as a function of back-gate voltage from the upstream and downsteam edge states. **(d-f)** show the same experiments data with (a-c) at B = −1.7 T.

At B = 1 T, the three-terminal measurements performed in the MLG device are shown in Figs. S5(a)-(c). As discussed in the main text, the AR signals start to appear for ν = 14

[denoted by arrows in Figs. S5(a)-(c)], a smaller value than for the BLG device, as the width W ~ 270 nm of the interface for the Nb−MLG device was narrower than for the Nb−BLG device (W ~ 360 nm), suffering less intervalley scattering at the junction interface. Reducing W helps to sustain the coherence of electron-hole pairs at the Andreev edge states until the final Andreev-reflected quasiparticles escape at the end of the Nb−MLG junction. But at ν = 22, the narrowed channel width may have acted as a disorder to the channels for higher filling factors, inducing slightly reduced QH conductance. In the Fig. S5(c), we reduce the magnetic length by increasing B field to see the clear QH plateaus of high filling factors. Now, G_U is gradually enhanced by the AR as the conducting modes of edge states were piled up by increasing V_{BG}. Whereas R_D did not show a negative resistance at the high filling factors in the incompressible states. We suppose that the AR probability of MLG device was much lower than the BLG device (ΔG ~ 14 % for zero bias at B = 0 T in the MLG device) which acted as a major obstacle to obtain the negative resistance. But the resistance dip structures from the proximity effects in Δ_{Nb} ~ 462 μeV (calculated at B = −1.7 T) still exist at the high filling factors in both the upstream and downstream sides of the QH edge states [see Figs. S5(e) and (f), respectively].

6. *I*-*V* characteristics without a superconducting electrode in monolayer graphene device (T = 0.16 K, B = 1.7 T)

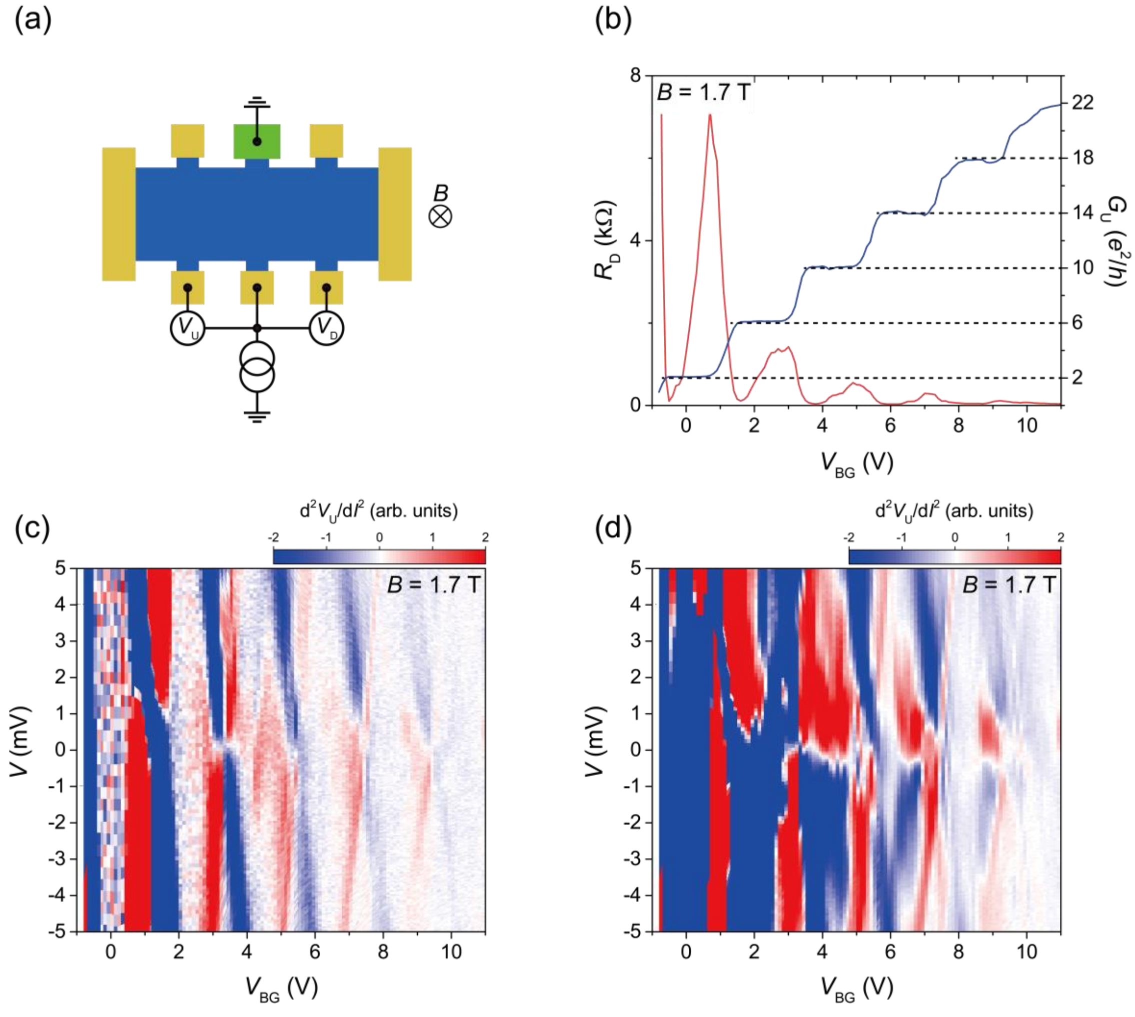

Figure S6 | Three-terminal *I*-*V* characteristics without a superconducting electrode. (a) A schematic diagram for measurement configuration. **(b)** Zero-bias conductance (blue) and resistance (red) from upstream and downstream sides of the Andreev edge states at B = 1.7 T. **(c), (d)** Colour maps for d^2V/dI^2 from V_U and V_D, respectively.

As a counter example, we took the similar three-terminal measurements on the same MLG device in main text, but in both the upstream and downstream edge states of the normal Au electrodes in the opposite side of the MLG sheet at B = 1.7 T. Figure S6(a) shows the schematic diagram of the measurement configuration. Since the Andreev pairs injected from the superconductors pass through the electrode reservoirs and travel a distance, significantly longer than the original measurement configuration, the pairs eventually lose their coherence or recombined before they reach the voltage probes. Therefore, only the trivial QH signals were obtained as shown in Fig. S6(b). Figures S6(c) and (d) show the colour maps of d^2V/dI^2 from the upstream and downstream voltage probes. The strong peak-dip structures from the QH edge states are sustained in the high filling factors, showing that there were no AR resistance dip signals within the gap energy of Nb.

References

1 Wang, L. *et al.* One-dimensional electrical contact to a two-dimensional material. *Science* **342**, 614 (2013).

2 Ben Shalom, M. *et al.* Quantum oscillations of the critical current and high-field superconducting proximity in ballistic graphene. *Nat. Phys.* **12**, 318-322 (2016).

3 Velasco, J. *et al.* Transport measurement of Landau level gaps in bilayer graphene with layer polarization control. *Nano Letters* **14**, 1324-1328 (2014).

4 Kurganova, E. V. *et al.* Quantum Hall activation gaps in bilayer graphene. *Solid State Communications* **150**, 2209-2211 (2010).
